# Supplementary material for: COZOID: contact zone identifier for visual analysis of protein-protein interactions
Source: BMC Bioinformatics. 2018 Apr 6;19:125. doi: 10.1186/s12859-018-2113-6 (PMC5889581; doi:10.1186/s12859-018-2113-6)
Supplement: Supplementary file 2 — Software build. Executable binary file of the software tool. (ZIP 118,784 kb) [file 12859_2018_2113_MOESM2_ESM.zip › cozoid/java/docs/beansbinding-1.2.1-doc.zip!/org/jdesktop/el/class-use/ELException.html]

Uses of Class org.jdesktop.el.ELException (Beans Binding)


---


|  |  |  |
| --- | --- | --- |
| **Overview** \| **Package** \| **Class** \| **Use** \| **Tree** \| **Deprecated** \| **Index** \| **Help** | |  |
| PREV   NEXT | **FRAMES**    **NO FRAMES**     **All Classes** |  |


---


## **Uses of Class org.jdesktop.el.ELException**

| Packages that use ELException | |
| --- | --- |
| **org.jdesktop.el** | Customized version of EL for Beans Binding; not for general use. |
| **org.jdesktop.el.impl** | Customized version of EL for Beans Binding; not for general use. |
| **org.jdesktop.el.impl.lang** | Customized version of EL for Beans Binding; not for general use. |
| **org.jdesktop.el.impl.parser** | Customized version of EL for Beans Binding; not for general use. |
| **org.jdesktop.el.impl.util** | Customized version of EL for Beans Binding; not for general use. |

Uses of ELException in org.jdesktop.el

| Subclasses of ELException in org.jdesktop.el | |
| --- | --- |
| `class` | `MethodNotFoundException`             Thrown when a method could not be found while evaluating a `MethodExpression`. |
| `class` | `PropertyNotFoundException`             Thrown when a property could not be found while evaluating a `ValueExpression` or `MethodExpression`. |
| `class` | `PropertyNotWritableException`             Thrown when a property could not be written to while setting the value on a `ValueExpression`. |

| Methods in org.jdesktop.el that throw ELException | |
| --- | --- |
| `Expression.Result` | `ValueExpression.getResult(ELContext context, boolean trackResolvedObjects)` |

Uses of ELException in org.jdesktop.el.impl

| Methods in org.jdesktop.el.impl that throw ELException | |
| --- | --- |
| `MethodInfo` | `MethodExpressionLiteral.getMethodInfo(ELContext context)` |
| `MethodInfo` | `MethodExpressionImpl.getMethodInfo(ELContext context)`             Evaluates the expression relative to the provided context, and returns information about the actual referenced method. |
| `Expression.Result` | `ValueExpressionImpl.getResult(ELContext context, boolean trackResolvedObjects)` |
| `java.lang.Class` | `ValueExpressionImpl.getType(ELContext context)` |
| `java.lang.Object` | `ValueExpressionImpl.getValue(ELContext context)` |
| `java.lang.Object` | `MethodExpressionLiteral.invoke(ELContext context, java.lang.Object[] params)` |
| `java.lang.Object` | `MethodExpressionImpl.invoke(ELContext context, java.lang.Object[] params)`             Evaluates the expression relative to the provided context, invokes the method that was found using the supplied parameters, and returns the result of the method invocation. |
| `boolean` | `ValueExpressionImpl.isReadOnly(ELContext context)` |
| `void` | `ValueExpressionImpl.setValue(ELContext context, java.lang.Object value)` |

Uses of ELException in org.jdesktop.el.impl.lang

| Methods in org.jdesktop.el.impl.lang that throw ELException | |
| --- | --- |
| `static int` | `ELSupport.compare(java.lang.Object obj0, java.lang.Object obj1)` |
| `MethodExpression` | `ExpressionBuilder.createMethodExpression(java.lang.Class expectedReturnType, java.lang.Class[] expectedParamTypes)` |
| `static Node` | `ExpressionBuilder.createNode(java.lang.String expr)` |
| `ValueExpression` | `ExpressionBuilder.createValueExpression(java.lang.Class expectedType)` |
| `static boolean` | `ELSupport.equals(java.lang.Object obj0, java.lang.Object obj1)` |
| `static void` | `ELSupport.throwUnhandled(java.lang.Object base, java.lang.Object property)` |
| `void` | `ExpressionBuilder.visit(Node node)` |

Constructors in org.jdesktop.el.impl.lang that throw ELException

`ExpressionBuilder(java.lang.String expression,
ELContext ctx)`

Uses of ELException in org.jdesktop.el.impl.parser

| Methods in org.jdesktop.el.impl.parser that throw ELException | |
| --- | --- |
| `void` | `SimpleNode.accept(NodeVisitor visitor)` |
| `void` | `Node.accept(NodeVisitor visitor)` |
| `MethodInfo` | `SimpleNode.getMethodInfo(EvaluationContext ctx, java.lang.Class[] paramTypes)` |
| `MethodInfo` | `Node.getMethodInfo(EvaluationContext ctx, java.lang.Class[] paramTypes)` |
| `MethodInfo` | `AstValue.getMethodInfo(EvaluationContext ctx, java.lang.Class[] paramTypes)` |
| `MethodInfo` | `AstIdentifier.getMethodInfo(EvaluationContext ctx, java.lang.Class[] paramTypes)` |
| `java.lang.Class` | `SimpleNode.getType(EvaluationContext ctx)` |
| `java.lang.Class` | `Node.getType(EvaluationContext ctx)` |
| `java.lang.Class` | `BooleanNode.getType(EvaluationContext ctx)` |
| `java.lang.Class` | `AstValue.getType(EvaluationContext ctx)` |
| `java.lang.Class` | `AstString.getType(EvaluationContext ctx)` |
| `java.lang.Class` | `AstNull.getType(EvaluationContext ctx)` |
| `java.lang.Class` | `AstNot.getType(EvaluationContext ctx)` |
| `java.lang.Class` | `AstNegative.getType(EvaluationContext ctx)` |
| `java.lang.Class` | `AstLiteralExpression.getType(EvaluationContext ctx)` |
| `java.lang.Class` | `AstInteger.getType(EvaluationContext ctx)` |
| `java.lang.Class` | `AstIdentifier.getType(EvaluationContext ctx)` |
| `java.lang.Class` | `AstFunction.getType(EvaluationContext ctx)` |
| `java.lang.Class` | `AstFloatingPoint.getType(EvaluationContext ctx)` |
| `java.lang.Class` | `AstEmpty.getType(EvaluationContext ctx)` |
| `java.lang.Class` | `AstDynamicExpression.getType(EvaluationContext ctx)` |
| `java.lang.Class` | `AstDeferredExpression.getType(EvaluationContext ctx)` |
| `java.lang.Class` | `AstCompositeExpression.getType(EvaluationContext ctx)` |
| `java.lang.Class` | `AstChoice.getType(EvaluationContext ctx)` |
| `java.lang.Class` | `ArithmeticNode.getType(EvaluationContext ctx)` |
| `java.lang.Object` | `SimpleNode.getValue(EvaluationContext ctx)` |
| `java.lang.Object` | `Node.getValue(EvaluationContext ctx)` |
| `java.lang.Object` | `AstValue.getValue(EvaluationContext ctx)` |
| `java.lang.Object` | `AstTrue.getValue(EvaluationContext ctx)` |
| `java.lang.Object` | `AstString.getValue(EvaluationContext ctx)` |
| `java.lang.Object` | `AstPlus.getValue(EvaluationContext ctx)` |
| `java.lang.Object` | `AstOr.getValue(EvaluationContext ctx)` |
| `java.lang.Object` | `AstNull.getValue(EvaluationContext ctx)` |
| `java.lang.Object` | `AstNotEqual.getValue(EvaluationContext ctx)` |
| `java.lang.Object` | `AstNot.getValue(EvaluationContext ctx)` |
| `java.lang.Object` | `AstNegative.getValue(EvaluationContext ctx)` |
| `java.lang.Object` | `AstMult.getValue(EvaluationContext ctx)` |
| `java.lang.Object` | `AstMod.getValue(EvaluationContext ctx)` |
| `java.lang.Object` | `AstMinus.getValue(EvaluationContext ctx)` |
| `java.lang.Object` | `AstLiteralExpression.getValue(EvaluationContext ctx)` |
| `java.lang.Object` | `AstLessThanEqual.getValue(EvaluationContext ctx)` |
| `java.lang.Object` | `AstLessThan.getValue(EvaluationContext ctx)` |
| `java.lang.Object` | `AstInteger.getValue(EvaluationContext ctx)` |
| `java.lang.Object` | `AstIdentifier.getValue(EvaluationContext ctx)` |
| `java.lang.Object` | `AstGreaterThanEqual.getValue(EvaluationContext ctx)` |
| `java.lang.Object` | `AstGreaterThan.getValue(EvaluationContext ctx)` |
| `java.lang.Object` | `AstFunction.getValue(EvaluationContext ctx)` |
| `java.lang.Object` | `AstFloatingPoint.getValue(EvaluationContext ctx)` |
| `java.lang.Object` | `AstFalse.getValue(EvaluationContext ctx)` |
| `java.lang.Object` | `AstEqual.getValue(EvaluationContext ctx)` |
| `java.lang.Object` | `AstEmpty.getValue(EvaluationContext ctx)` |
| `java.lang.Object` | `AstDynamicExpression.getValue(EvaluationContext ctx)` |
| `java.lang.Object` | `AstDotSuffix.getValue(EvaluationContext ctx)` |
| `java.lang.Object` | `AstDiv.getValue(EvaluationContext ctx)` |
| `java.lang.Object` | `AstDeferredExpression.getValue(EvaluationContext ctx)` |
| `java.lang.Object` | `AstCompositeExpression.getValue(EvaluationContext ctx)` |
| `java.lang.Object` | `AstChoice.getValue(EvaluationContext ctx)` |
| `java.lang.Object` | `AstBracketSuffix.getValue(EvaluationContext ctx)` |
| `java.lang.Object` | `AstAnd.getValue(EvaluationContext ctx)` |
| `java.lang.Object` | `SimpleNode.invoke(EvaluationContext ctx, java.lang.Class[] paramTypes, java.lang.Object[] paramValues)` |
| `java.lang.Object` | `Node.invoke(EvaluationContext ctx, java.lang.Class[] paramTypes, java.lang.Object[] paramValues)` |
| `java.lang.Object` | `AstValue.invoke(EvaluationContext ctx, java.lang.Class[] paramTypes, java.lang.Object[] paramValues)` |
| `java.lang.Object` | `AstIdentifier.invoke(EvaluationContext ctx, java.lang.Class[] paramTypes, java.lang.Object[] paramValues)` |
| `boolean` | `SimpleNode.isReadOnly(EvaluationContext ctx)` |
| `boolean` | `Node.isReadOnly(EvaluationContext ctx)` |
| `boolean` | `AstValue.isReadOnly(EvaluationContext ctx)` |
| `boolean` | `AstIdentifier.isReadOnly(EvaluationContext ctx)` |
| `boolean` | `AstDynamicExpression.isReadOnly(EvaluationContext ctx)` |
| `boolean` | `AstDeferredExpression.isReadOnly(EvaluationContext ctx)` |
| `static Node` | `ELParser.parse(java.lang.String ref)` |
| `void` | `SimpleNode.setValue(EvaluationContext ctx, java.lang.Object value)` |
| `void` | `Node.setValue(EvaluationContext ctx, java.lang.Object value)` |
| `void` | `AstValue.setValue(EvaluationContext ctx, java.lang.Object value)` |
| `void` | `AstIdentifier.setValue(EvaluationContext ctx, java.lang.Object value)` |
| `void` | `AstDynamicExpression.setValue(EvaluationContext ctx, java.lang.Object value)` |
| `void` | `AstDeferredExpression.setValue(EvaluationContext ctx, java.lang.Object value)` |
| `void` | `NodeVisitor.visit(Node node)` |

Uses of ELException in org.jdesktop.el.impl.util

| Methods in org.jdesktop.el.impl.util that throw ELException | |
| --- | --- |
| `static java.beans.PropertyDescriptor` | `ReflectionUtil.getPropertyDescriptor(java.lang.Object base, java.lang.Object property)` |

---


|  |  |  |
| --- | --- | --- |
| **Overview** \| **Package** \| **Class** \| **Use** \| **Tree** \| **Deprecated** \| **Index** \| **Help** | |  |
| PREV   NEXT | **FRAMES**    **NO FRAMES**     **All Classes** |  |


---
